# The *Brucella* Cell Envelope

**DOI:** 10.1146/annurev-micro-032521-013159

**Published:** 2023-04-27

**Authors:** Melene A. Alakavuklar, Aretha Fiebig, Sean Crosson

**Affiliations:** Department of Microbiology and Molecular Genetics, Michigan State University, East Lansing, Michigan, USA;

**Keywords:** *Alphaproteobacteria*, zoonosis, intracellular pathogen, gram negative

## Abstract

The cell envelope is a multilayered structure that insulates the interior of bacterial cells from an often chaotic outside world. Common features define the envelope across the bacterial kingdom, but the molecular mechanisms by which cells build and regulate this critical barrier are diverse and reflect the evolutionary histories of bacterial lineages. Intracellular pathogens of the genus *Brucella* exhibit marked differences in cell envelope structure, regulation, and biogenesis when compared to more commonly studied gram-negative bacteria and therefore provide an excellent comparative model for study of the gram-negative envelope. We review distinct features of the *Brucella* envelope, highlighting a conserved regulatory system that links cell cycle progression to envelope biogenesis and cell division. We further discuss recently discovered structural features of the *Brucella* envelope that ensure envelope integrity and that facilitate cell survival in the face of host immune stressors.

## OVERVIEW

### The Gram-Negative Envelope

The cell envelope separates the cytoplasm of a cell from the exterior environment. It determines cell shape; harbors macromolecular complexes that generate usable forms of energy to power motility and growth; and directly interacts with features of the surrounding environment, including plant and animal hosts (70, 111). The envelope of gram-negative bacteria is particularly interesting because it comprises two structurally distinct lipid bilayers with a thin periplasm and peptidoglycan cell wall in the space between them (111). The ability of micron-sized bacterial cells to maintain this structurally complex, didermic envelope while growing and dividing is a remarkable feat.

Our understanding of the gram-negative envelope is primarily based on studies of the class *Gammaproteobacteria*, specifically *Enterobacteriaceae* such as *Escherichia coli*. However, gram-negative bacteria are a varied group of organisms that exhibit remarkable diversity in the chemical and structural makeup of their envelopes. In recent years, cellular and molecular studies of the *Alphaproteobacteria* have revealed conserved molecular features that distinguish the envelope of this class. Species in the *Alphaproteobacteria* thus provide useful comparative models when considering which molecular processes of gram-negative envelope biogenesis and homeostasis are general, and which are specific to a particular phylogenetic group (38).

### *Brucella*: A Brief Introduction

The *Alphaproteobacteria* include species that inhabit diverse niches, including the plant rhizosphere and phyllosphere; freshwater, marine, and soil ecosystems; and mammalian and insect hosts (11). *Brucella* is perhaps the most notorious genus in this class. *Brucella abortus* and *Brucella melitensis* were first identified in the late nineteenth century as the etiologic agents of contagious abortion in cows and Malta fever in humans, respectively (9, 18). These intracellular pathogens cause a disease now known as brucellosis, which remains among the most widespread zoonoses globally (32, 96). The *Brucella* genus is genetically monomorphic, and it has been proposed to be monospecific (128). However, *Brucella* species cluster into classifiable phylogenetic groups that align with animal host range and select molecular and physiologic characteristics, including differences in the structure and chemical makeup of the cell envelope (90). In this review we provide an overview of the *Brucella* cell envelope and highlight recent advances in the study of *Brucella* spp. envelope structure, function, and regulation.

## DEVELOPMENTAL REGULATION OF *BRUCELLA* CELL ENVELOPE BIOGENESIS

### The CtrA Regulatory Network

**Figure 1:**
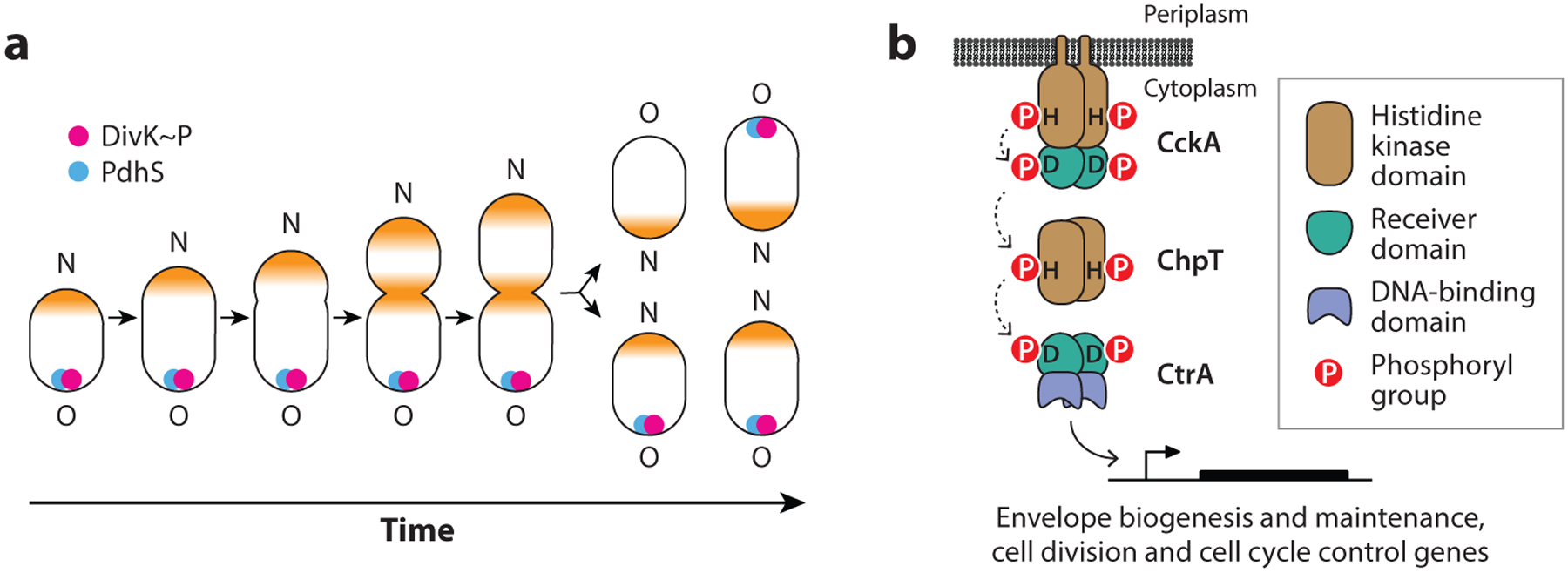
(*a*) *Brucella* spp., like other *Rhizobiales*, exhibit unipolar growth from the new cell pole. As the cell cycle progresses, cell growth shifts from being exclusively polar to being at both the pole and the nascent division site (growth sites colored *orange*). The *Brucella* developmental regulators, PdhS and DivK, exhibit dynamic polar localization to the inner membrane as a function of cell cycle. The new (N) pole and old (O) cell pole are marked. (*b*) Model of the CckA-ChpT-CtrA phosphorelay. The histidine kinase CckA autophosphorylates on a conserved histidine residue and transfers a phosphoryl group to a conserved aspartic acid residue on its C-terminal receiver domain. CckA~P transfers a phosphoryl group to the ChpT phosphotransferase, which can subsequently transfer this phosphoryl group to the receiver domain of CtrA. CtrA~P is a DNA-binding response regulator that modulates transcription of genes controlling cell polarity, division, and intracellular survival.

When viewed from afar, *Brucella* looks like any other gram-negative bacteria: it possesses a phospholipid bilayer inner membrane (IM), an outer membrane with a phospholipid inner leaflet and a lipopolysaccharide (LPS) outer leaflet, and a peptidoglycan cell wall in the periplasmic space between. Close inspection of single cells has provided strong evidence for an asymmetric growth mechanism. Specifically, peptidoglycan labeling experiments in *B. abortus* demonstrated that new cell envelope material is synthesized strictly from the new cell pole produced immediately after cell division (17, 64). At later stages of the cell cycle, a small growth zone appears at the nascent division site in *B. abortus* (101, 103, 127) (Figure 1a). Asymmetry in the biogenesis of new cell material during growth is reflected in asymmetric subcellular organization of key protein regulators of *Brucella* cell cycle and development. For example, the essential polar development sensor histidine kinase, PdhS, colocalizes with the response regulator DivK to the old cell pole during the *B. abortus* cell cycle; DivK localization requires the conserved aspartyl phosphorylation site in its receiver domain. Upon division, polarly localized PdhS is asymmetrically inherited by the mother cell; the newborn cell later acquires PdhS at its old pole, approximately 30 min after division (56) (Figure 1a). The developmental processes regulated by PdhS and DivK are integrated into a large regulatory network that is conserved across *Alphaproteobacteria* and that ensures the molecular machinery required to build new cell material is (*a*) localized to the appropriate polar location and (*b*) activated at the appropriate time during the cell cycle (77, 95, 125, 136). This elaborate molecular control system comprises multiple sensor histidine kinases, response regulators, diguanylate cyclases, and phosphodiesterases, all of which influence the expression, phosphorylation, and stability of the essential DNA-binding response regulator, CtrA. It can be reasonably argued that CtrA is the central molecular player of cell cycle and cell development regulation in many *Alphaproteobacteria*, including *Brucella* (98).

Like other response regulators, the activity of *Brucella* CtrA as a transcription factor is controlled by its phosphorylation at a single aspartic acid residue. The phosphorylation state of the CtrA protein is directly regulated by a multiprotein phosphorelay comprising the transmembrane sensor histidine kinase CckA and the histidine phosphotransferase ChpT (134) (Figure 1b). Chromatin immunoprecipitation sequencing (ChIP-seq) studies of *B. abortus* CtrA have demonstrated that CtrA directly binds the promoter regions of dozens of genes involved in cell division, peptidoglycan cell wall metabolism, outer membrane biogenesis, and biosynthesis of LPS (43). The importance of CtrA as a direct regulator of a diverse set of cell envelope proteins is clearly evident in studies of depletion and temperature-sensitive mutants of *B. abortus* (43, 134). Shifting conditional *ctrA* mutant strains to restrictive conditions resulted in reduced levels of select outer membrane proteins (OMPs) and the formation of filamentous branched cells, consistent with a defect in polar cell development and division. Branched cells were also observable in the mammalian intracellular niche upon depletion of *ctrA* expression; this phenotype was correlated with reduced intracellular viability (43). Attenuation of a conditional *ctrA* mutant in an in vitro infection model is expected, given that *B. abortus* cell cycle progression is intimately tied to intracellular trafficking in the endosomal compartments (33).

### A Role for Cyclic Diguanylate?

The concentration and asymmetric localization of the second messenger cyclic-di-GMP (c-di-GMP) directly control the phosphorylation state and stability of CtrA in *Caulobacter crescentus* (77, 79, 112). A direct role for this signaling molecule in regulation of the *Brucella* CtrA pathway has not been shown, but there is evidence that c-di-GMP metabolizing enzymes play a role in *Brucella* envelope biology. A screen of annotated *B. melitensis* diguanylate cyclases and phosphodiesterases in a heterologous *Vibrio* system identified at least three active enzymes, including the phosphodiesterase PdeA (also known as BpdA) (97). *B. melitensis pdeA* mutants were attenuated in vitro and in vivo (67, 97), and deletion of *B. abortus* and *B. melitensis pdeA* resulted in cell rounding, with cells becoming shorter and wider (67, 101). This shape phenotype was independent of any phosphodiesterase activity the enzyme may have in cells, as a catalytically deficient *pdeA*^E742A^ mutant retained wild-type morphology (101). Peptidoglycan analysis using fluorescent d-amino acid (HADA) labeling provided evidence that PdeA is required for localizing the site of peptidoglycan insertion to the pole (101). This result is consistent with studies of a PdeA homolog in *Sinorhizobium meliloti* known as RgsP, which functions at the membrane to control peptidoglycan composition (108).

Though PdeA is an important regulator of envelope biology in *Brucella*, a role for the c-di-GMP molecule per se has not been defined. Studies of diverse bacteria have shown that c-di-GMP levels in the cytoplasm control the transition from motile to sessile behavior via a variety of molecular mechanisms (31, 105). *Brucella* spp. contain assorted flagellar genes and pseudogenes (26, 36) but have long been classified as nonmotile bacteria (109). A *B. melitensis* strain was reported to elaborate a sheathed flagellum under select growth conditions (44), but efforts to identify actively motile cells among the classical *Brucella* species (*B. abortus*, *B. melitensis*, *B. ovis*, *B. suis*, *B. canis*, and *B. neotomae*) have been unsuccessful to our knowledge. However, several new *Brucella* species have been isolated from a range of animals (133), including amphibian isolates that are clearly motile and elaborate flagella and pilus-like structures from their envelopes (3). Whether c-di-GMP influences motility in these newly identified flagellated and motile *Brucella* species is not known, but it seems likely considering reported connections between *pdeA* and transcription from flagellar promoters in *B. melitensis* (97). Future efforts to define a specific role(s) for c-di-GMP as an effector of envelope processes in *Brucella* may be informed by studies in related free-living rhizobial species (e.g., *S. meliloti*) where c-di-GMP levels are linked to motility, polysaccharide production, and cell wall biosynthesis (71).

## THE *BRUCELLA* OUTER MEMBRANE

The outer membrane is the interface between *Brucella* cells and their environment. This asymmetrical lipid bilayer contains primarily LPS in the outer leaflet and phospholipids in the inner leaflet. As an intracellular pathogen, *Brucella* must withstand a variety of host assaults, including compounds that disrupt membranes, such as antimicrobial peptides. Relative to a panel of *Enterobacteriaceae*, including *E. coli*, *Brucella* spp. outer membranes are more resistant to envelope stressors including polymyxin B, melittin, EDTA, and lysozyme (84). This enhanced resistance has been attributed to particular OMPs, distinct chemical features of core lipid A, and the density of O-polysaccharides of the LPS leaflet of the outer membrane, which will be discussed below.

### Outer Membrane Proteins

The outer membrane is likely the best-studied of the *Brucella* envelope layers because it contains many antigens, including a diverse array of OMPs, that invoke host immune responses (47). Focused effort to characterize *Brucella* OMPs over the past several decades (129) have been motivated in part by the fact that OMPs can—in some cases—confer protective immunity, facilitate serologic classification of strains, and be developed as vaccines. We now know that *Brucella* spp. encode multiple families of OMPs, including many heat-stable porins and lipoproteins. A large body of literature on *Brucella* OMP content, structure, and immunology has been covered in previous reviews (24, 52, 106), and we encourage readers to consult these for additional information and additional perspectives on this topic.

### Targeting and Assembly: Lol, Bam, and Tam

**Figure 2:**
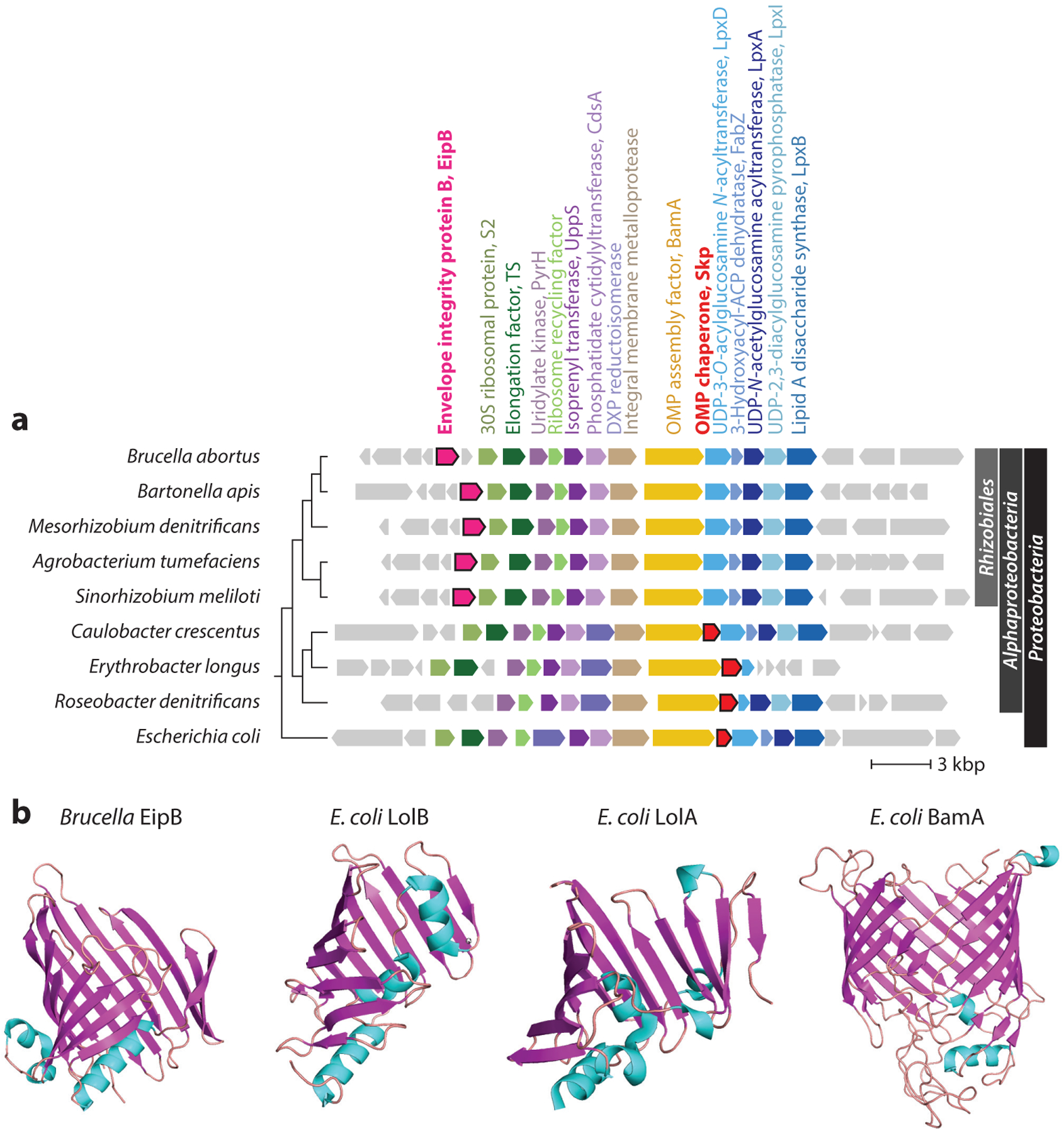
(*a*) Phylogenetic distribution and synteny of the envelope integrity protein B (*eipB*) genomic region in *Proteobacteria*; gene neighborhood is anchored on *bamA*. *Brucella eipB* is part of a highly conserved cell envelope gene cluster in the *Proteobacteria* that includes genes involved in outer membrane protein assembly (*bamA*), undecaprenyl phosphate biosynthesis, phospholipid synthesis (*cdsA*), lipopolysaccharide synthesis (*lpxDAIB*), and translation (*ef-ts*). *eipB* (*pink*) is conserved in the *Rhizobiales* group of the *Alphaproteobacteria* (phylogenetic classifications on the *right*). Skp is present outside the *Rhizobiales*. Phylogenetic tree is based on BamA protein sequence (*left*). (*b*) Crystal structures of *Brucella* EipB (PDB: 6NTR), *Escherichia coli* LolB (PDB: 1IWM), *E. coli* LolA (PDB: 1IWL), and *E. coli* BamA (PDB: 5OR1). Abbreviation: PDB, Protein Data Bank.

The mechanism by which lipid-modified OMPs (i.e., lipoproteins) become localized to the *Brucella* outer membrane remains an open question. In gram-negative bacteria, the lipoprotein localization (Lol) pathway comprises a set of proteins that traffic outer membrane lipoproteins across the periplasm to their final address (54). However, the gene encoding the LolB protein, which is necessary for lipoprotein trafficking in many gram-negative bacteria, is absent in the *Alphaproteobacteria*, including *Brucella* (52, 92). It has been proposed that LolA may serve the functions of both LolA and LolB in clades where LolB is absent (118), though this hypothesis remains untested in *Brucella*. Recent efforts to define the functions of uncharacterized *B. abortus* proteins involved in envelope stress responses identified a periplasmic domain of unknown function protein (DUF1849) conserved in a subset of *Alphaproteobacteria*—primarily *Rhizobiales*—that is now named EipB (61). The *eipB* chromosomal locus exhibits synteny homology across the *Rhizobiales* with genes that function in membrane and cell wall synthesis, LPS synthesis, and outer membrane protein assembly. The overall organization of this genomic region is highly conserved in *Proteobacteria* (Figure 2a), and the association of *eipB* with this locus supports a functional role for *eipB* in cell envelope biology. EipB comprises 14 antiparallel β strands, organized in a cylindrical, spiral-like shape, with three α-helical connector segments. Though EipB has no clear structural homologs in the Protein Data Bank, its β-barrel architecture resembles those of *E. coli* LolA and LolB, and the OMP assembly proteins TamA and BamA (Figure 2b). Deletion of *eipB* resulted in sensitivity to compounds that disrupt the integrity of the cell envelope and compromised *B. abortus* infection in a murine model of disease (61). These recent results support a functional role for EipB in determining cell envelope integrity, but it is not known whether EipB assumes a LolB-like function in lipoprotein trafficking, whether it functions as an OMP chaperone or assembly factor, or whether it has another role in the *Brucella* cell envelope.

In addition to the Lol system, there are other molecular systems that ensure *Brucella* appropriately assembles OMPs into the outer membrane. Among these is the β-barrel assembly machine (BAM) complex, which facilitates protein folding into the outer membrane (81). The genus *Brucella* encodes homologs of the BamA, BamD, and BamE proteins and is missing the BamB and BamC proteins present in *E. coli* and other *Gammaproteobacteria*. Our application of Bayesian or HMM gene essentiality algorithms (34, 35) to published transposon sequencing (Tn-Seq) data from *B. abortus* 2308 (61, 62) and *B. ovis* 25840 (126) provides evidence that *bamE*, *bamD*, and *bamA* are essential in both species. Anwari and colleagues (4) identified a BAM complex component, BamF (DUF3035), that is restricted to the *Alphaproteobacteria*, though sequence models in the InterPro-Pfam database (15) indicate that BamF/DUF3035 is absent in the genus *Brucella*. The composition of the BAM complex varies across gram-negative bacteria, and it is not known what proteins—if any—fulfill the functional roles of BamB, BamC, and BamF in *Brucella*. The fact that the periplasmic β-barrel protein, EipB, is encoded from a locus proximal to *bamA* suggests that it could function with the BAM complex in OMP assembly (Figure 2a). Notably, the OMP chaperone Skp is encoded adjacent to BamA in many *Proteobacteria*; this gene is absent from *Rhizobiales* cataloged in InterPro. The presence of *eipB* in this conserved cell envelope cluster (instead of *skp*) in *Rhizobiales* raises the possibility that EipB and Skp have analogous chaperoning roles.

The outer membrane translocation and assembly module (TAM) is a system that is evolutionarily related to the BAM complex and plays a key role in the assembly of select OMPs (58). The system comprises two protein components: the integral OMP TamA, which is related to BamA, and the inner membrane protein TamB. Studies in *B. suis* have shown that the TamB homolog, known as MapB, controls translocation of a subset of OMPs to the outer membrane (13). A *B. suis mapB* deletion mutant had disrupted localization of select OMPs, a cell division/morphology defect, and was sensitive to multiple envelope-disrupting compounds, including polymyxin B, Triton X-100, and lysozyme (13). *mapB/tamB* deletion in *B. suis* (13) and *B. melitensis* (135) resulted in strain attenuation in infection models, and *B. abortus* strains lacking either *tamA* or *tamB* were attenuated in a mouse macrophage infection model (116). A specific role for the TAM system during infection is supported by proteomic analyses showing that the steady-state level of TamB protein in *B. abortus* (BAB1_0046/Omp160) was enhanced ~20–100-fold just 3 h after macrophage infection and remained high over a two-day infection time course (75).

### The Outer Membrane–Peptidoglycan Connection

**Figure 3:**
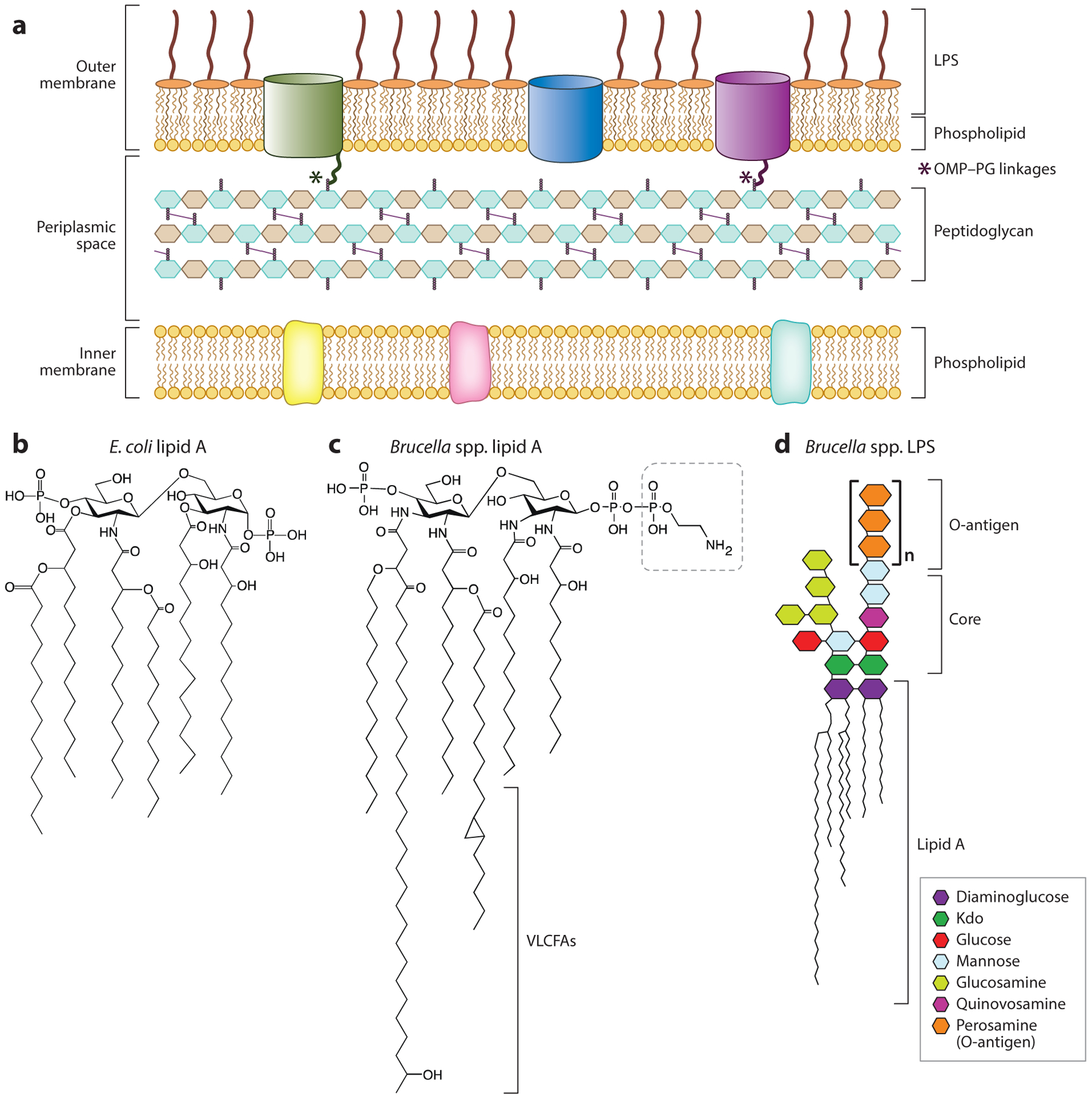
(*a*) Overview of the envelope layers of *Brucella*. Notably, a subset of *Brucella* outer membrane proteins are covalently linked to the peptidoglycan cell wall via a conserved sequence at the protein N terminus. (*b*) Chemical structure of *Escherichia coli* lipid A (100). (*c*) Chemical structure of *Brucella* spp. lipid A showing VLCFA tails and pyrophosphorylethanolamine modification of diaminoglucose backbone (outlined with dashed-line box). Structure adapted from model and data presented in References 20 and 29. (*d*) *Brucella* smooth LPS structure showing lipid A, core oligosaccharide, and O-polysaccharide (O-antigen), based on models and data presented in References 41, 72, and 117. Abbreviations: Kdo, 3-deoxy-d-*manno*-2-octulosonic acid; LPS, lipopolysaccharide; OMP, outer membrane protein; PG, peptidoglycan; VLCFA, very-long-chain fatty acid.

In *E. coli* and related bacteria, the hyperabundant outer membrane lipoprotein Lpp is covalently linked to the peptidoglycan (8, 16, 57), which provides important structural support for the envelope. *Brucella* and other related *Rhizobiales* lack *lpp*, but biochemical evidence for covalent interactions between *Brucella* spp. OMPs and the peptidoglycan cell wall (25, 51) has been long discussed. Recent work by Godessart and colleagues (49) has demonstrated that multiple OMPs in *B. abortus* and the related rhizobial species *Agrobacterium tumefaciens* are covalently linked to the peptidoglycan via a conserved alanyl-aspartyl motif at the protein N terminus (Figure 3a). Like the covalent linkage of *E. coli* Lpp to peptidoglycan, this linkage in *Brucella* is catalyzed by l,d-transpeptidases. This result explains long-noted observations of peptidoglycan linkages to OMPs in *Brucella* and provides new biochemical and structural understanding of *Brucella* envelope integrity. Notably, several other bacteria lacking Lpp have now been shown to anchor the outer membrane to peptidoglycan in a similar fashion to *Brucella* (107).

## LIPOPOLYSACCHARIDE

LPS, the primary lipid of the outer leaflet of the outer membrane, is a major pathogen-associated molecular pattern (PAMP) recognized by the innate immune system (120). Accordingly, *Brucella* LPS is a highly studied molecule that has been well-reviewed (106, 117, 137), and we encourage readers to consult these reviews for more information on this topic. Here we provide an abridged overview of distinctive features of *Brucella* LPS structure and biosynthesis.

### LPS Structure

The structure of *Brucella* spp. LPS has chemical features that distinguish this envelope component from the highly inflammatory LPS of enteric bacteria (Figure 3b) and that enable these pathogens to evade host recognition by the innate immune system (10, 28). Canonically, LPS consists of a hexa-acylated disaccharide called lipid A, which is linked to the core oligosaccharides. In species with smooth LPS, repeating O-polysaccharide units decorate the core polysaccharide (Figure 3). The lipid A portion of *Brucella* LPS consists of diaminoglucose sugars linked to C16–C18 fatty acids and very-long-chain fatty acids (VLCFAs), including a significant proportion of 27-OH-28:0 (91) (Figure 3c). This unusual feature of *Brucella* lipid A is restricted to the alpha-2 subgroup of *Proteobacteria*, and it may enable lipid A to extend through the outer membrane outer leaflet into the inner leaflet (Figure 3a), potentially increasing the structural integrity of the outer membrane (12). The level of VLCFAs on lipid A is controlled in part by the inner membrane–bound BacA protein in both *B. abortus* and the related rhizobial species *S. meliloti* (39), suggesting that this unique LPS feature can be regulated. Relative to other gram-negative bacteria, *Brucella* lipid A is a poor stimulant of the mammalian inflammatory response (37). The core oligosaccharide of *Brucella* spp. lipid A contains two 3-deoxy-d-*manno*-2-octulosonic acid (Kdo) sugars (89) linked to diaminoglucose, one of which has a branched linkage to a chain of glucose, mannose, and glucosamine (48) (Figure 3d). There is evidence that this branched core structure shields lipid A from recognition by the host innate immune sensor TLR4 (28) and thereby contributes to the reduced inflammatory capacity of *Brucella* LPS.

Among the classical *Brucella* species (132), the best-described difference in cell envelope composition is the presence or absence of O-polysaccharide, also known as O-antigen (117). *Brucella* spp. O-antigen consists primarily of N-formylated perosamine (50) (Figure 3d) of two major linkage forms: A (from *B. abortus*) and M (from *B. melitensis*) (72, 87). While *Brucella* spp. typically elaborate an O-antigen and are considered to have smooth LPS (S-LPS), *B. ovis* and *B. canis* harbor genetic mutations that result in an inability to synthesize O-antigen and have what is therefore considered naturally rough LPS (R-LPS). O-antigen is an important virulence determinant in *Brucella* (76), yet *B. ovis* and *B. canis* cause disease in their primary hosts, sheep and dogs, respectively. The pathogenicity of these rough strains in their primary hosts is notable because rough mutants of naturally smooth strains have large defects in intracellular growth and replication and can be cytotoxic to host macrophages.

The structure of O-antigen from the numerous new *Brucella* species identified in recent years (133) remains largely undefined, but several strains in the recently identified BO2 clade of *Brucella* are missing genes in the *wbk* region that are required for S-LPS synthesis. Experimental analyses of a BO2 strain showed that it produced an S-LPS (138) not composed of *N*-formyl-perosamine (130). In the place of the *wbk* genes, this strain contained genes for biosynthesis of a rhamnose-based O-antigen; BO2 S-LPS had biochemical properties that were distinct from those of the classical *Brucella* species (130). Future analyses of LPS structure across the expanded *Brucella* clade will likely illuminate new and diverse chemical features of *Brucella* LPS.

### Spatial Control of LPS Biosynthesis in *Brucella*

An elaborate transport system ensures proper addressing of LPS to the outer membrane in gram-negative bacteria (115). In *B. abortus*, the biosynthesis and elaboration of LPS on the surface of the cell is spatially controlled. *B. abortus* has both R-LPS and S-LPS patches on its envelope that can be visualized by immunofluorescence (127). Insertion of new LPS material occurs unipolarly at the new pole of the cell (generated immediately after division), where new cell wall material is also added (127). The inner membrane LPS biosynthesis proteins LptB, LptC, LptF, and LptG are similarly localized to the new pole. There are conflicting reports of the spatial distribution of the outer membrane LPS translocase, LptD. Fluorescent protein fusions to LptD showed a polar localization pattern in wild-type *B. abortus* (123) consistent with the localization of LptBCFG, while staining by immunogold provides evidence that LptD is localized across the cell surface (110).

### Periplasmic Factors Linked to LPS

Recent studies have identified previously uncharacterized domain of unknown function (DUF) proteins that are secreted to the periplasm and have roles in cell envelope processes. The periplasmic β-barrel protein EipB (61), which may function in OMP targeting, is described above. Studies of *Brucella* RomA and EipA provide evidence that these periplasmic proteins have a functional connection to LPS.

The RomA protein is a member of the DUF3126 superfamily, which is restricted to the *Alphaproteobacteria*; in *B. abortus* this protein is secreted to the periplasm (123). A *B. abortus romA* deletion strain (Δ*romA*) had multiple envelope defects including altered LPS composition: Δ*romA* had a higher proportion of S-LPS, with longer O-polysaccharide chains than the wild type (123). This LPS phenotype was correlated with altered localization of a fluorescent protein fusion to the LPS translocase, LptD. Specifically, the Δ*romA* strain showed diffuse localization of LptD across the cell, while LptD was polarly localized in the wild type. Localization of the O-polysaccharide flippase, RfbD (Bab1_0543), was not altered in Δ*romA*. The precise function of RomA remains undefined, but it seems likely that this protein functions in the periplasm to coordinate LPS synthesis.

**Figure 4:**
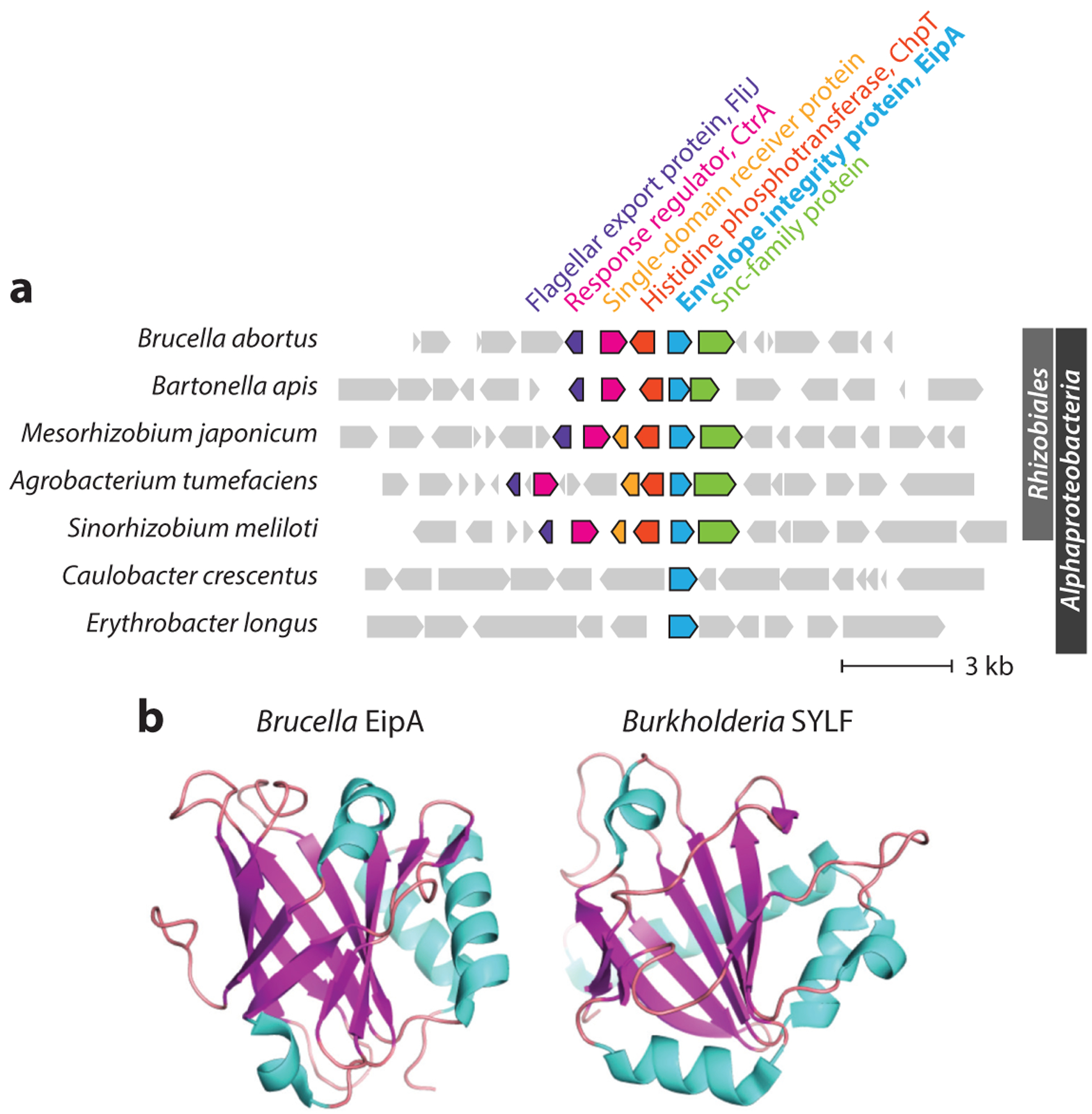
(*a*) Phylogenetic distribution and synteny of the envelope integrity protein A (*eipA*) genomic region in *Proteobacteria*; gene neighborhood is anchored on *eipA*. In *Brucella* and other *Rhizobiales*, *eipA* is part of a genetic locus that encodes the essential cell cycle regulatory proteins CtrA and ChpT. (*b*) Crystal structures of *Brucella* EipA (PDB 5UC0) and a *Burkholderia* SYLF domain protein (PDB 7OFN). Abbreviation: PDB, Protein Data Bank.

The *Brucella* EipA protein is a member of the DUF1134 protein superfamily, which is largely restricted to the *Alphaproteobacteria* (Figure 4a). In the *Rhizobiales*, *eipA* is encoded in the neighborhood of *ctrA* and *chpT* (Figure 4a), and its expression is directly controlled by CtrA (62). EipA is secreted to the periplasm, and deletion of *B. abortus eipA* resulted in sensitivity to envelope stressors including EDTA, SDS, and ampicillin (62). As would be expected for a strain with a general envelope defect, Δ*eipA* was attenuated in macrophage and mouse infection models. The molecular function of EipA in the periplasm has not been determined, but a screen for genes that are synthetically lethal with *eipA* deletion in *B. abortus* showed that disruption of O-polysaccharide synthesis genes is not tolerated in the absence of *eipA* (62). Congruent with this genetic analysis in *B. abortus*, *eipA* is essential in the naturally rough species *B. ovis*, which lacks the O-polysaccharide. A high-resolution crystal structure of EipA revealed a novel protein fold (62) composed of 10 antiparallel β strands, with β1–β5 and β8–β10 forming a small β barrel (Figure 4b). Though EipA interaction partners have not been defined, a recent solution NMR structure of the lipid/phosphoinositide-binding SYLF domain protein BPSL1445 revealed significant structural similarity to *Brucella* EipA, suggesting that EipA is a lipid-interacting protein (99).

## *BRUCELLA* PHOSPHOLIPIDS

Phospholipids are a key molecular component of the cell envelope, and *Brucella* has interesting lipid membrane features that merit discussion. Phosphatidylethanolamine (PE) is the dominant phospholipid in most gram-negative bacteria, but *Brucella* membranes contain comparatively low PE and a high proportion of phosphatidylcholine (PC), a phospholipid usually associated with eukaryotic membranes (45, 113, 122). PE synthesis in *B. abortus* is apparently dispensable; deletion of phosphatidylserine synthase (PssA), which catalyzes the first step in PE synthesis, is compensated by increased production of PC and ornithine lipids (OLs) (19). It has been proposed that an elevated PC level may provide a mechanism to mask pathogens, including *Brucella*, from their mammalian hosts (2, 27, 30). This idea is supported by studies of the phospholipase A1, BveA, of *B. melitensis*. Analysis of lipid extracts of a *bveA* mutant revealed elevated levels of PE. Enhanced PE levels were associated with higher susceptibility to polymyxin B and reduced survival in in vitro and in vivo infection models (66).

The notable skew in the PC:PE ratio in *Brucella* spp. relative to other gram-negative bacteria has motivated studies of the mechanism of PC biosynthesis. PC can be synthesized through one of two pathways: the methylation pathway and the choline pathway. In the methylation pathway, PmtA mediates successive methylation of phosphatidylethanolamine, using *S*-adenosylmethionine (SAM) as a methyl donor. In the choline pathway, the enzyme Pcs synthesizes PC from choline and cytidine diphosphate-diacylglycerol precursors (113). The *B. abortus* genome contains both *pcs* and *pmtA* homologs, but genetic analysis of Δ*pcs* and Δ*pmtA* mutants showed that only *pcs* was required for normal growth and synthesis of PC in complex medium (27, 30). However, both Δ*pcs* and Δ*pmtA* mutants were attenuated for replication in the mouse spleen at different stages of infection, and a Δ*pcs* Δ*pmtA* double mutant (*a*) had a larger growth defect in defined medium than single mutants and (*b*) was more attenuated in mouse spleen than either single mutant (30). These results provide evidence that both pathways to PC are operational in *B. abortus* and that the nutritional environment influences the requirement for these genes. A related series of experiments on PC synthesis in *B. melitensis* 16M extracts led to the conclusion that Pcs was the sole route to PC production (85), while the conclusion based on experiments in *B. abortus* was that PC was synthesized from host-derived choline exclusively via the choline (Pcs) pathway (27). Acquisition of choline from the host is consistent with a report that *B. abortus* requires the high-affinity choline transporter ChoXWV for PC synthesis when choline concentrations are low (59). Polymorphisms in the SAM-binding site of PmtA across the *Brucella* genus likely influence the enzymatic routes to PC production at the species level and may explain why select *B. abortus*, *B. melitensis*, and *B. suis* strains have differing requirements for *pcs* and *pmtA* in vitro and in vivo (5).

In addition to PC and PE, the *Brucella* envelope also contains a significant fraction of OLs (94, 122). Prior to studies in *Brucella*, OL synthesis had been characterized in *S. meliloti*, where two genes, *olsA* and *olsB*, are required for OL synthesis (46, 131). *Brucella* contains homologs of these genes and synthesizes OL through a two-step pathway (94). Deletion of OL synthesis did not impact membrane permeability, susceptibility to antimicrobial peptides, or infection in an in vitro model (94). Moreover, a *B. abortus* strain lacking OLs did not induce an inflammatory response in mice that differed from wild type. These results suggest that *Brucella* OLs have little impact on *B. abortus* envelope stress resistance, infection, or host immunity. Nonetheless, the abundance of OLs in *Brucella* membranes suggests these molecules have an important role in some context.

## THE PEPTIDOGLYCAN CELL WALL

As a gram-negative bacterium grows, it builds new peptidoglycan cell wall in the periplasmic space. At the point of division, the cell must cleave and reanneal the peptidoglycan wall that surrounds it. This highly complex process requires exquisite spatiotemporal coordination of a multitude of proteins on a submicron scale and has been the subject of intense study for many years (104). *E. coli*, *C. crescentus*, and many other model bacteria grow laterally and assemble the division site (i.e., the divisome) at the center of the cell after a certain period of growth. The *Rhizobiales* (including *Brucella*) grow by budding, a process in which new peptidoglycan material is added to one cell pole (17) (Figure 1a). The mechanism by which *Brucella* and other *Rhizobiales* localize the peptidoglycan biosynthesis machinery to one pole and maintain cell envelope integrity during the process of new cell addition and division remains largely undefined (124).

Penicillin-binding proteins (PBPs) are critical for peptidoglycan cell wall synthesis, and a transposon screen of *B. abortus* in complex medium uncovered Pbp1a (Bab1_0932) and FtsI as the only two essential PBPs of the seven encoded in the genome (116). As expected, this study also identified numerous divisome (*fts*) and cell division (*tol-pal*) genes as essential for growth. Screening this same transposon library in macrophages identified a set of genes that were conditionally essential in the intracellular niche. Among these was the histidine biosynthesis gene, *hisB*, which exhibited a surprising cell chaining/division defect inside mammalian cells resulting from uncleaved peptidoglycan at the cell division site (103). The *hisB* division phenotype could be suppressed by overexpression of either DipM or CdlP, each of which contains a peptidoglycan-binding LysM domain and is predicted to function as a metallopeptidase. This targeted suppressor approach thus identified two putative peptidoglycan metallopeptidases involved in *Brucella* cell division, though the exact connection between histidine metabolism, peptidoglycan cleavage, and division remains undefined. Cell wall metabolism and cell division are a relatively new area of investigation in *Brucella*, and future studies are certain to elucidate interesting connections between central metabolism and division.

## CONSERVED CELL ENVELOPE REGULATION SYSTEMS

When infecting a host, the *Brucella* cell must withstand many host-derived stressors to survive. It is the cell envelope that meets this complex host assault. Accordingly, the composition of the envelope is highly regulated by multiple stress response systems. In this section, we discuss select *Brucella* envelope regulators that are conserved in the *Alphaproteobacteria*.

### The General Stress Response System

**Figure 5:**
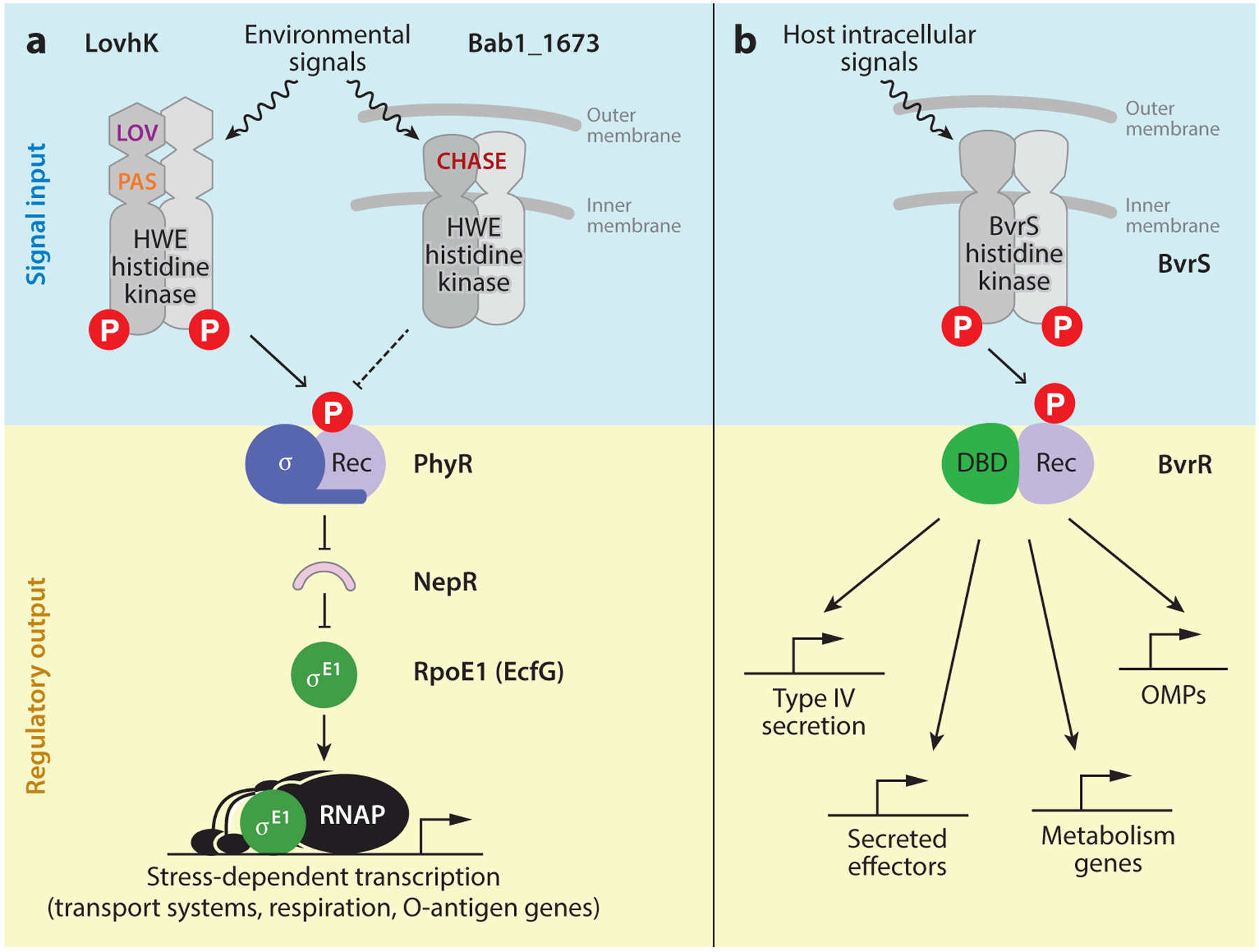
(*a*) The GSR signaling pathway in *Alphaproteobacteria* is activated by environmental signals and integrates features of two-component and sigma factor–dependent regulation of gene expression. Studies of this pathway in *Brucella abortus* support a model in which phosphorylation of PhyR is controlled by two HWE-family sensor kinases: LovhK and Bab1_1673. PhyR phosphorylation promotes its binding to NepR, which releases σ^E1^ to control transcription of select cell envelope genes that influence in vitro stress survival and host infection. The LOV and PAS sensory domains of the cytoplasmic GSR-activating kinase, LovhK, are labeled. The periplasmic CHASE family sensor domain of the transmembrane GSR-repressive kinase, Bab1_1673, is labeled. Solid lines indicate a direct interaction; dashed lines indicate interactions that may be direct or indirect. (*b*) *Brucella* spp. BvrR/BvrS is a conserved, archetypal two-component system that is a critical regulator of infection. In response to detection of intracellular signals during infection, the histidine kinase BvrS phosphorylates BvrR, which then regulates transcription of a suite of cell envelope and metabolic genes important for intracellular survival and replication. Abbreviations: DBD, DNA-binding domain; GSR, general stress response; REC, REC, receiver domain; RNAP, RNA polymerase; OMP, outer membrane protein.

The general stress response (GSR) system of *Alphaproteobacteria* regulates large-scale changes in gene expression that confer resistance to a range of environmental stressors (40, 42). Activation of the *B. abortus* GSR involves stress-dependent phosphorylation of the anti-anti-sigma protein PhyR (68, 69, 119). PhyR phosphorylation promotes its binding to the anti-σ^E1^ protein, NepR (80), thereby releasing the alternative sigma factor σ^E1^ (also known as *ecfG*) to regulate transcription of dozens of genes (Figure 5a). Studies in *B. abortus* and several other *Alphaproteobacteria* provide evidence that multiple HWE-family sensor kinases (60) coordinately regulate PhyR phosphorylation and GSR activation (Figure 5a), which may explain how this regulatory system is able to respond to such a diverse range of stress conditions (40, 42).

Deletion of GSR regulatory genes in *B. abortus* and *B. melitensis* results in a variable replication/survival defect in mice depending on the particular gene deletion and the genetic background of the animal host (53, 68, 69). The transcription of numerous *B. abortus* membrane transport systems is strongly activated by σ^E1^, including an RND-family efflux system, a CrcB-family transporter, ABC-type transporters, and a major facilitator family transporter (68). Thus *B. abortus* remodels the transport capabilities of its envelope when the GSR system is activated. In addition, transcription of the *cydABX* high-affinity terminal oxidase genes and the O-polysaccharide biosynthesis gene, *pgm*, is activated by σ^E1^, as are multiple hypothetical transmembrane DUF proteins. In *B. melitensis*, deletion of the GSR-activating kinase *lovhK* is reported to activate expression of the type IV secretion system (*virB*) and select flagellar genes (53). Together, these studies provide evidence that the GSR system is a major regulator of the *Brucella* cell envelope.

### The BvrR-BvrS Envelope Homeostasis System

The BvrR-BvrS two-component regulatory system is conserved in the *Alphaproteobacteria*, where it has important functions in host-bacteria interactions (22, 83). In *Brucella*, this system is a key determinant of infection in in vitro and in vivo models (78, 114). *B. abortus bvrR-bvrS* directly and indirectly regulates the type IV secretion system (86), contributes to homeostasis of membranes, and regulates lipid A acylation and the expression of periplasmic and outer membrane proteins (55, 74, 82) (Figure 5b). The general cell envelope defect of strains harboring mutations in this two-component system is evident in the fact that deletion of the envelope integrity protein *eipA* is synthetically lethal with *bvrR* deletion in *B. abortus* (62). Recent ChIP-seq analysis of *B. abortus* BvrR demonstrates that this regulator not only directly binds the promoter regions of type IV secretion genes (*virB*), genes for secreted effectors, and genes that function in multiple aspects of cell envelope homeostasis but also binds promoters of multiple genes with roles in central metabolism (102). This study provides evidence for a broader regulatory role for the BvrR-BvrS system in *Brucella* cell envelope biology and metabolism than has been previously appreciated.

### Ros/MucR

Like the BvrR-BvrS system, the zinc finger transcription factor Ros/MucR is conserved in the *Alphaproteobacteria* (1, 7, 23, 65), where it has an established role in virulence gene regulation and plant-rhizobia interactions (6, 14, 23). Deletion of this regulator in multiple *Brucella* species results in alterations in cell envelope properties (21, 88, 121) that, in *B. melitensis*, lead to sensitivity to a range of cell envelope stressors in vitro. Studies in *B. melitensis* further provide evidence that MucR regulates modification of lipid A core and regulates transcription of flagellar genes via the flagellar regulator protein FtcR (88). *Brucella mucR* can functionally complement an *S. meliloti mucR* deletion mutant (88), providing an additional example of how a conserved set of regulatory proteins underpins host-microbe interactions in *Alphaproteobacteria*.

## CONCLUSIONS AND OUTLOOK

*Brucella* spp. are important animal pathogens that have been studied for over a century. Since their discovery, we have gained significant understanding of animal host responses to infection and the *Brucella* genetic factors that determine infection and pathogenesis. Molecular components of the cell envelope play a crucial role in host interactions, and subtle differences in cell envelope composition or regulation likely contribute to the fascinating animal host preferences exhibited by the highly related *Brucella* species. Deciphering the genetic basis of host preference is an interesting and exciting area of investigation that may be advanced by combining pangenome analyses with omics-based approaches to analyze cell envelope lipids, polysaccharides, and proteins.

*Brucella* has emerged as a powerful comparative model for the investigation of cell cycle, polar cell development, and cell division in gram-negative bacteria. The development of fluorescent d-amino acids for study of peptidoglycan synthesis was instrumental in demonstrating that cell growth in *Brucella* is unipolar (73), and this tool will be useful as the community works to decipher the molecular mechanism of polar cell growth in *Brucella* and other *Rhizobiales*. Select genes for other well-studied polar surface structures in *Alphaproteobacteria*, such as unipolar polysaccharide (UPP) (63, 93), are conserved in *Brucella*. It is not known whether *Brucella* spp. elaborate a UPP, but it seems likely that cell cycle–regulated polar envelope structures—perhaps including UPP— contribute to the interesting link between the developmental state of the *Brucella* cell and the process of host infection (33).

## Abbreviations

LPS: lipopolysaccharide
S-LPS: smooth lipopolysaccharide
R-LPS: rough lipopolysaccharide
PE: phosphatidylethanolamine
PC: phosphatidylcholine
